# Vesicular CLC chloride/proton exchangers in health and diseases

**DOI:** 10.3389/fphar.2023.1295068

**Published:** 2023-11-07

**Authors:** Alessandra Picollo

**Affiliations:** ^1^ Institute of Biophysics, National Research Council, Genova, Italy; ^2^ RAISE Ecosystem, Genova, Italy

**Keywords:** vCLC, genetic diseases, exchangers, structures, function

## Abstract

Chloride is one of the most abundant anions in the human body; it is implicated in several physiological processes such as the transmission of action potentials, transepithelial salt transport, maintenance of cellular homeostasis, regulation of osmotic pressure and intracellular pH, and synaptic transmission. The balance between the extracellular and intracellular chloride concentrations is controlled by the interplay of ion channels and transporters embedded in the cellular membranes. Vesicular members of the CLC chloride protein family (vCLCs) are chloride/proton exchangers expressed in the membrane of the intracellular organelles, where they control vesicular acidification and luminal chloride concentration. It is well known that mutations in CLCs cause bone, kidney, and lysosomal genetic diseases. However, the role of CLC exchangers in neurological disorders is only now emerging with the identification of pathogenic *CLCN* gene variants in patients with severe neuronal and intellectual dysfunctions. This review will provide an overview of the recent advances in understanding the role of the vesicular CLC chloride/proton exchangers in human pathophysiology.

## 1 Introduction

The CLC chloride transporting protein family includes four chloride channels (ClC-1, ClC-2, ClC-Ka, and ClC-Kb) expressed in the plasma membrane and five chloride/proton exchangers (from ClC-3 to ClC-7) expressed in the membrane of intracellular compartments. They are implicated in several physiological processes, including muscle contraction, transepithelial salt reabsorption, acidification of intracellular compartments, and cellular homeostasis (Jentsch and Pusch, 2018). The vCLCs can be divided into two subclasses depending on their sequence and functional similarity. ClC-3, ClC-4, and ClC-5 form the first subclass with almost 80% sequence identity. The renal ClC-5 is expressed in early endosomes, while ClC-3 and ClC-4 localize in sorting and late endosomes. ClC-6 and ClC-7, which share 45% of sequence identity, constitute the second subclass. ClC-6 resides in late endosomes, while ClC-7 localizes in lysosomes and osteoclasts, where it is implicated in bone resorption (Jentsch and Pusch, 2018). All CLCs share a similar overall dimeric architecture, with a large transmembrane domain and a cytosolic region containing two CBS (cystathionine β synthase) domains (Accardi, 2015). Recent studies have demonstrated that ClC-4 is more stable when it forms heterodimers with ClC-3 (Weinert et al., 2020).

The relevance of vCLC proteins is shown by their involvement in severe pathologies, such as neurodegeneration (ClC-3 and ClC-6) (Stobrawa et al., 2001; Polovitskaya et al., 2020), global developmental delay (ClC-3) (Duncan et al., 2021), intellectual disability combined with epilepsy, various psychiatric conditions (ClC-4) (Veeramah et al., 2013; Hu et al., 2016; Palmer et al., 2018), lysosomal storage diseases (ClC-6 and ClC-7) (Kasper, 2005; Poet et al., 2006; He et al., 2021), osteopetrosis without or with neurodegeneration (ClC-7) (Kornak et al., 2001; Kasper, 2005), and Dent’s disease (ClC-5) (Lloyd et al., 1996; Piwon et al., 2000).

With the advent of next-generation sequencing analysis, many new genetic defects are emerging. Despite the difficulty in understanding these data, it was possible to identify new pathophysiological roles for vCLCN genes, providing an extensive picture of the role of vCLCs in human physiology. This review aims to provide an overview of the similarities and differences in structural, functional, and physiological properties of vCLCs.

## 2 Structures

**FIGURE 1:**
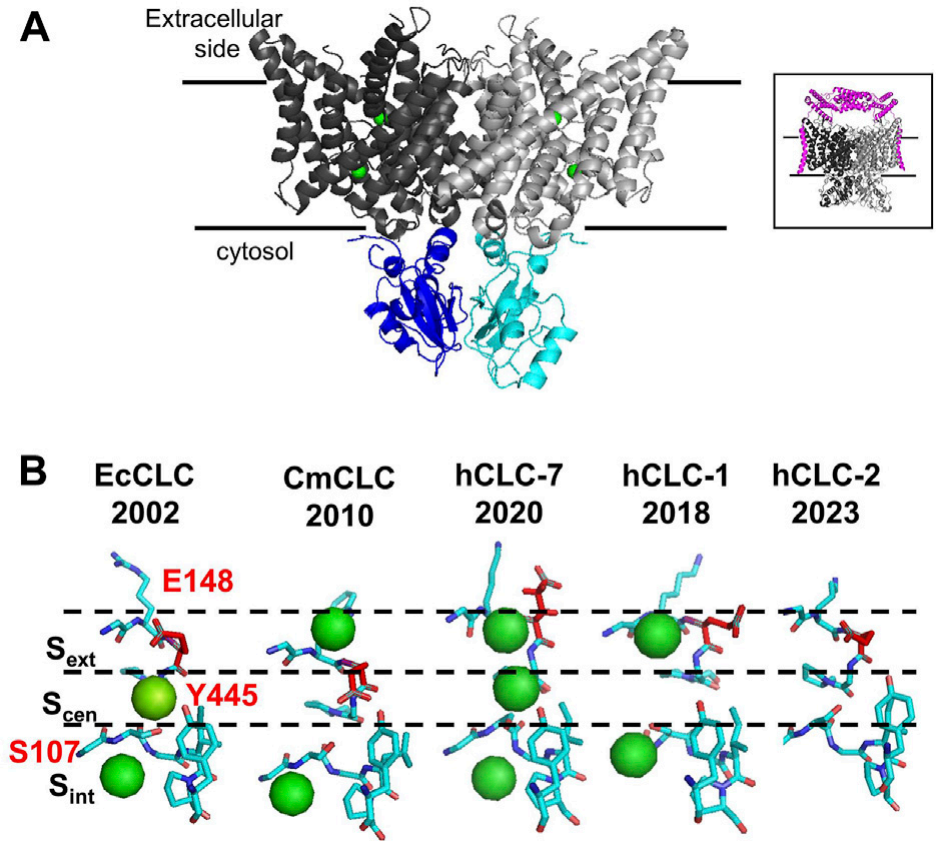
Structural properties of vCLC proteins. **(A)** Structure of the algae vCLC homolog CmClC, viewed within the membrane. Transmembrane domains are colored in dark and light gray and cytosolic domains are colored in blue and cyan. Green spheres correspond to chloride ions in the permeation pathway. In the inset, the structure of hClC-7 in complex with OSTM1 is shown in magenta. **(B)** View of the four conformations of the ion binding region captured in the corresponding protein structures solved until now. The gating glutamate is highlighted in red. Cl^−^ ions detected by cryo-EM are shown as green spheres. [PDB ID: EcClC (1OTS), CmClC (3ORG), hClC-7 (7JM6), hClC-1 (6QVC), and hClC-2 (7xF5)].

The development of cryo-EM technology allowed the resolution of the three-dimensional structures of several mammalian CLC channels and exchangers, but ClC-3, ClC-4, and ClC-5 structures are still missing. CLCs are homodimers with two identical subunits, each containing a large transmembrane domain (TMd, Figure 1A, in gray) and a cytosolic domain (CTd Figure 1A in cyan and blue). Each TMd is formed by 18 alpha helices (from helix A to R) tilted with respect to the plane of the biological membranes; some helices partially span the membranes, forming short loops within the protein. The cytosolic domain is composed of two cystathionin-β-synthase (CBS) domains containing two putative ATP binding sites. In vCLCs, each monomer contains a chloride and a proton permeation pathway. The two pathways converge toward the extracellular exit, sharing a common external gate, but they diverge toward the intracellular exit. The chloride permeation pathway is highly conserved between CLC channels and exchangers: three chloride binding sites, S_ext_, S_cen_, S_int_, have been identified. Chloride ions bound to S_ext_ and S_int_ are in contact with the extracellular and intracellular solutions, respectively. Conversely, the ion bound to S_cen_, located inside the protein, is coordinated by the side chain of the “gating glutamate” (Glu_ext_), whose protonation/de-protonation state determines the opening or closing of the external gate, and by the side chains of a serine (Ser_cen_) and a tyrosine (Tyr_cen_). The side chain of Glu_ext_ has been captured in two closed and two open configurations (Figure 1B). In the outward closed state, the side chain of Glu_ext_ occludes the extracellular chloride exit or occupies the S_ext_ (Figure 1B, EcCLC (Dutzler et al., 2002) and hCLC-2 (Ma et al., 2023)) or the S_cen_ (Figure 1B, CmCLC (Feng et al., 2010)). In the outward open state, the side chain of Glu_ext_ points or moves up toward the extracellular space (Figure 1B, hCLC-7 (Schrecker et al., 2020)) or toward the protein dimer interface (Figure 1B hCLC-1 (Park and MacKinnon, 2018)). The neutralization of Glu_ext_ transforms vCLC exchangers into pure chloride conductors (Picollo and Pusch, 2005; Scheel et al., 2005) and leaves CLC channels constitutively open (Traverso et al., 2003; Zuniga et al., 2004). In protein exchangers, to avoid free ion diffusion, an internal gate is necessary. Two models have been proposed. The first suggests that Cl^−^ ions have to overcome a kinetic barrier to move from S_cen_ to S_int_ or *vice versa* (Feng et al., 2012). In the second model, the chloride internal gate is physically constituted by the side chain of Tyr_cen_ (Accardi et al., 2006). Indeed, the H^+^ translocation pathway is not well defined. In addition to Glu_ext_, a second glutamate called “proton glutamate,” Glu_int_, which is closed to the cytosol and is located far away from the chloride permeation pathway, has been proposed as the internal proton acceptor. However, it is unclear how protons can travel the approximately 15 Å hydrophobic region separating Glu_ext_ from Glu_int_. Molecular dynamic simulations revealed the formation of transient water wires that connect the two H^+^ sites (Han et al., 2014; Lee et al., 2016), suggesting a water-mediated H^+^ transport mechanism. Unfortunately, direct functional experiments to prove the role of water wires in proton transport across CLCs are challenging. The comparison of all solved structures shows minimal local structural differences and the absence of water molecules, which could be due to a lack of sufficient experimental resolution. Indeed, NMR, biochemical, and electrophysiological studies suggest that during the transport cycle, global structural rearrangements occur (Abraham et al., 2015; Basilio et al., 2015), which may be consistent with conformational states where water wires can be formed.

The structure of hClC-7 in complex with its accessory protein, Ostm1 (Schrecker et al., 2020; Zhang et al., 2020) (Figure 1 A, inset), has revealed few specific structural features. First, the large highly glycosylated luminal region of Ostm1 is positioned above the luminal side of ClC-7 as a lid to protect ClC-7 from lysosomal degradation, and the unique transmembrane domain of Ostm1 interacts with ClC-7 helices facing the membrane lipid environment. Surprisingly, a phosphatidylinositol-3-phosphate (PI3P) molecule, a constituent of lysosomal membranes, has been found bound to the external surface of ClC-7 at the interface between TMd and Ctd (Schrecker et al., 2020). It has been shown that PI(3,5)P2 inhibits ClC-7 activity, but it fails in the case of the Y715C ClC-7 mutation (Leray et al., 2022), which causes lysosomal storage diseases without osteopetrosis (Nicoli et al., 2019; Leray et al., 2022). It is not known if PI3P molecules can interact with other vCLCs and modulate their transport activity.

## 3 Physiology and pathophysiology of vCLCs

**FIGURE 2:**
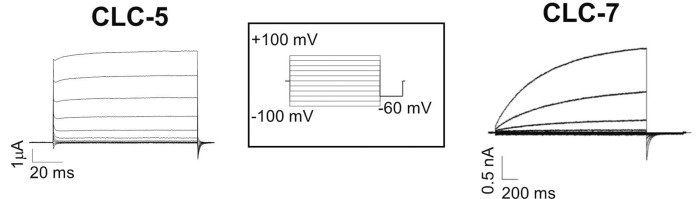
Typical recording of vCLC currents. Left two electrode voltage clamp currents recording of ClC-5 expressed in *Xenopus* oocytes. Right patch clamp currents recording of the plasma membrane ClC-7 construct. The stimulation voltage-clamp protocol is shown in the inset. Starting from the reversal potential, voltage steps were from −100 to 100 mV with increments of 20 mV for a duration that was protein-dependent (for 100 ms for ClC-5 and 2 s for ClC-7), and then −60 mV was applied.

Vesicular CLCs share common characteristics. First, they are mainly expressed in the vesicles of the endo-lysosomal pathway, in osteoclasts, in phagosomes, and in synaptic vesicles (Jentsch and Pusch, 2018). Together with the vacuolar ATPase proton pump (V-ATase), vCLCs are implicated in the regulation of the pH of intracellular organelles. Second, their biophysical properties are quite similar, but obtaining a complete functional characterization of all vCLCs took many years due to the difficulty of expressing them in plasma membranes. This limit was overcome by modification of the amino terminus or by using alternative splicing variants, as in the case of vClC-3 (Rohrbough et al., 2018) or by site-directed mutagenesis, as for vClC-7, where a leucine-based lysosomal sorting motif was identified and neutralized, partially redirecting vClC-7 to the plasma membrane (Stauber and Jentsch, 2010), or by the fusion of green fluorescence protein (GFP) to the N-terminus of vCLC-6 (Neagoe et al., 2010). vCLCs are electrogenic Cl^−^/H^+^ exchangers with a conserved stoichiometry of two chloride ions per one proton (Picollo and Pusch, 2005; Scheel et al., 2005; Graves et al., 2008; Neagoe et al., 2010; Leisle et al., 2011; Rohrbough et al., 2018). When vCLCs are expressed in the plasma membrane of *Xenopu*s oocytes or mammalian cells, they all show strongly outwardly rectifying currents. They activate at positive membrane potentials (Figure 2), but vClC-3-ClC-4 and ClC-5 show almost instantaneous activation (Picollo and Pusch, 2005; Duncan et al., 2021) (Figure 2, left), while vClC-6 and 7 show quite slow kinetics of activation (Leisle et al., 2011; Zifarelli et al., 2022) (Figure 2, right). These outward currents correspond to the movement of chloride ions out of intracellular vesicles, coupled with an influx of protons. However, analysis of mouse models suggests that the transmembrane proton gradient of intracellular vesicles drives an increase in luminal chloride concentration, reducing the membrane potential and facilitating vesicular acidification by V-ATPase (Pressey et al., 2010; Bose et al., 2021). Mathematical model simulations (Marcoline et al., 2016, Ishida, 2013 #5753) show that an electrogenic 2Cl^-^/H^+^ exchanger allows more efficient vesicular acidification by the V-ATPase pump than a simple chloride channel.

Third, *Clcn5*, *Clcn3*, *Clcn6,* and *Clcn7* knockout mice exhibit severe phenotypes resembling the severe forms of the corresponding diseases, with one exception: the *Clcn4* ^*−/−*^ mouse, which shows a milder phenotype without any neurodegeneration. In laboratory mice, the *CLCN4* gene is not located on the X chromosome as in humans. An animal model with conserved human chromosome localization might be more informative and necessary for proper *CLCN4* gene physiological investigation.

A common characteristic of *CLCN*-related disorders is a wide range of clinical symptoms, from lethal to almost asymptomatic, and a large number of pathogenic variants that cause several different functional consequences: impaired protein folding, altered protein localization, and changes in chloride and/or proton transport properties. This variety of functional alterations and clinical symptoms, combined with the limited number of patients carrying the same CLCN pathogenic variant, often represents a limit to a clear genotype-phenotype correlation.

### 3.1 ClC-5 and Dent’s disease

vClC-5 is expressed in the early endosomes of the epithelial cells of the proximal tube (PTCs) and in the intercalating cells of the distal nephron (Günther et al., 1998; Sakamoto et al., 1999). Although in lower abundance, ClC-5 was also found in the medullary thick ascending and thin descending limbs of Henle’s loop in the kidney (Devuyst et al., 1999). ClC-5, with the electrogenic vacuolar H^+^-ATPase (V-ATPase), contributes to the regulation of intra-endosomal pH and is involved in the protein uptake process in PTCs (Günther et al., 1998). Genetic defects in the *CLCN5* gene cause Dent’s disease type I (Thakker, 1997; Jentsch and Pusch, 2018), a rare genetic disorder characterized by low molecular weight proteinuria, hypercalciuria, and kidney stones, mainly due to impaired endocytosis. The most common *CLCN5* genetic defects are frameshift mutations that frequently result in protein misfolding and missense mutations causing loss of function (Gianesello et al., 2020). Moreover, knockout mice show altered renal endosomal acidification and reduced endocytosis in PTCs (Günther et al., 2003; Silva et al., 2003). If *CLCN5* is converted in mice into a simple Cl^−^ conductor (*Clcn5* ^*unc/unc*^), the endosomal acidification is maintained, but the clinical phenotypes resemble those of patients affected by Dent’s disease or those developed by knockout mice (Scheinman, 1998; Piwon et al., 2000; Wang et al., 2000), suggesting that chloride conductance alone without H^+^ coupling is not sufficient to support tubular endocytosis.

### 3.2 ClC-3 and ClC-4 and heterodimers

ClC-3 and ClC-4 are broadly found in many mammalian tissues, including the brain, heart, liver, kidney, pancreas, intestine, and skeletal muscle (Kawasaki et al., 1995; Stobrawa et al., 2001); they show a similar relative abundance in the brain and muscle (Weinert et al., 2020), and both localize in the endosomes. ClC-3 has also been found in osteoclasts (Okamoto et al., 2008), and its localization and role in synaptic vesicles are still controversial (Maritzen et al., 2008; Weinert et al., 2020; Bose et al., 2021). In addition, ClC-3 is expressed in the insulin granule, where it may be implicated in insulin secretion by promoting the acidification of insulin granules (Li et al., 2009). Even if the physiological roles of ClC-3 and ClC-4 are not completely clear yet, animal models and human genetic diseases provide useful information. Knockout *Clcn3* mice manifest severe neurodegeneration, leading to blindness and almost complete loss of the hippocampus in the first month of the mice’s life (Stobrawa et al., 2001; Weinert et al., 2020). A different *Clcn3* mouse model, in addition, shows signs of lysosomal storage disease (Yoshikawa et al., 2002). Differently from the *Clcn5* ^*unc/unc*^ mouse model, Weinert et al. recently showed that the *Clcn3* ^*unc/unc*^ mouse does not manifest a clear phenotype. In the same study, Weinert et al. observed a significant reduction in the level of ClC-4 protein in both *Clcn3* ^*−/−*^ mice and heterozygous *Clcn3* ^*+/−*^ mice. However, in *Clcn4* ^*−/−*^ mice, they did not observe any neurodegeneration or reduction in ClC-3 protein level. To verify if the reduction of ClC-4 contributes to severe neurodegeneration in *Clcn3* ^*−/−*^ mice, they generated two genotype combinations: *Clcn3* ^*−/−*^;*Clcn4* ^*−/−*^ and *Clcn3* ^*unc*^ *;Clcn4* ^*−/−*^. They found that *Clcn3* ^*unc/unc*^ *;Clcn4* ^*−/−*^ mice developed more severe neurodegeneration than *Clcn3* ^*−/−*^ mice but milder than *Clcn3* ^*−/−*^ *;Clcn4* ^*−/−*^. All this data demonstrates that ClC-4 forms stable heterodimers with ClC-3, suggesting that ClC-3 could partially compensate for the loss of ClC-4 and explaining the large variability of phenotype observed in patients carrying mutations on the *CLCN4* gene. The physiological relevance of vClC-3 and vClC-4 is also emerging from the pathogenic *CLCN3* and *CLCN4* variants that have been found in patients affected by global developmental delay, intellectual disability, and neurodevelopmental disorders (Veeramah et al., 2013; Hu et al., 2016; Palmer et al., 2018; Duncan et al., 2021; Guo et al., 2021; Xu et al., 2021; Palmer et al., 2022). Electrophysiological analysis of these variants has revealed new biophysical mechanisms, including the gain of function and shift in voltage dependence of current gating (Duncan et al., 2021; Palmer et al., 2022).

### 3.3 Endolysosomal ClC-6 and ClC-7 Cl^−^/H^+^ exchangers

ClC-6 is primarily expressed in the late endosomes of the nervous system, where it partially overlaps with ClC-7 (Brandt and Jentsch, 1995; Poet et al., 2006; Neagoe et al., 2010). *Clcn6* ^−/−^ knockout mice showed mild neuronal lysosomal storage disease (Poet et al., 2006), resembling the phenotype observed in two patients carrying ClC-6 heterozygous missense mutations (V580M and T628R) and affected by neuronal ceroid lipofuscinosis (NCL) (Poet et al., 2006). Instead, the neutralization of the gating glutamate with alanine (E200A) is responsible for West syndrome. When the E200A mutant is heterologously expressed *in vitro*, it alters the autophagy lysosomal pathway (He et al., 2021) and converts ClC-6 into a pure chloride conductor (Neagoe et al., 2010). A fourth missense mutation (Y553C) was found in three unrelated children who had severe developmental delays with pronounced generalized hypotonia, respiratory insufficiency, and variable neurodegeneration (Polovitskaya et al., 2020). Electrophysiological measurements revealed that Y553C induces a drastic change in ClC-6 ion transport properties, causing a pH-dependent gain of function (stronger at luminal acidic pH) (Polovitskaya et al., 2020; Zifarelli et al., 2022). Moreover, the expression of Y553C in various cell lines exhibited an alteration in cell morphology, with the formation of enlarged vesicles (Polovitskaya et al., 2020) resembling those observed in transfected cells with the ClC-7 missense mutation, Y715C, which causes severe lysosomal storage and albinism without osteopetrosis in humans (Nicoli et al., 2019). All these observations suggest that the alteration of the endosomal-lysosomal pathway might be responsible for neurodegeneration in both cases. Instead, a decrease in the total currents has been observed in the V580M mutant, indicating a predominantly negative impact in the heterozygous variant (Zifarelli et al., 2022). For the T628R mutation, no difference in electrical activity has been detected with respect to the WT. Similar results have been reported for pathogenic ClC-4 (Palmer et al., 2022), ClC-5 (Tang et al., 2016), and ClC-7 (Leisle et al., 2011) mutants that reside either within the intracellular domain or within loops exposed to the intra/extracellular space. Classical electrophysiological *in vitro* approaches will likely fail to identify the pathogenic mechanisms induced by these variants.

ClC-7 is broadly expressed, and primarily, it localizes in the lysosomes (Kornak et al., 2001). Recently, it has been shown that ClC-7 also localizes in mature phagosomes where ClC-7 is important for Cl^−^ accumulation and for phagosomal acidification (Wu et al., 2023). In the osteoclasts, through exocytosis, ClC-7 is inserted in the ruffled border of the reabsorption lacuna, a region in contact with the bone matrix and responsible for bone reabsorption (Lange et al., 2006). ClC-7 co-localizes with its accessory protein, Ostm1, to form a protein complex (Lange et al., 2006). Ostm1 shields un-glycosylated ClC-7 from degradation by lysosomal proteases (Lange et al., 2006; Schrecker et al., 2020; Zhang et al., 2020) and it is required for correct localization and function (Leisle et al., 2011). ClC-7 KO mice show severe osteopetrosis without changes in lysosomal acidification (Kornak et al., 2001; Kasper, 2005) but with a reduction in luminal [Cl^−^] (Weinert et al., 2010), and lysosomal storage disease, associated with retinal degeneration and severe neurodegeneration (Kornak et al., 2001; Kasper, 2005). Spontaneous Ostm1-deficient gray-lethal mice exhibit similar ClC-7 KO mice phenotype, and it mimics the severe human malignant autosomal recessive form of osteopetrosis (Chalhoub et al., 2003). *Clcn7* ^*unc/unc*^ mice, in which ClC-7 is converted into a pure chloride conductor, show a milder osteopetrosis phenotype with no changes in the fur color, but they develop lysosomal storage disease and neurodegeneration similar to *Clcn7* ^*−/−*^ mice (Weinert et al., 2010). A third mouse model, in which the transport activity of ClC-7 is abolished (*Clcn7* ^*td/td*^), manifests severe osteopetrosis as in *Clcn7* ^*−/−*^ mice but with less severe neurodegeneration (Weinert et al., 2014). Mutations in the *CLCN7* gene have been found in patients affected by autosomal recessive osteopetrosis with or without primary neurodegeneration (ARO) or by autosomal dominant osteopetrosis (ADOII) that show a quite large degree of severity, even when almost asymptomatic (Pangrazio et al., 2010; Bollerslev et al., 2013). Consistent with this heterogeneity of symptoms, electrophysiological investigations of several ClC-7 missense mutations causing diseases reveal a large variety of functional protein alterations, including impaired protein localization, complete or partial abolition of transport activity, gain of function, and accelerated kinetics of activation (Leisle et al., 2011; Nicoli et al., 2019; Di Zanni et al., 2021).

## 4 Conclusion

The review provides an overview of the structural similarity of vCLCs and presents recent advances in mice model investigations and functional analysis of pathogenic variants. It highlights that the variety of clinical symptoms and pathogenic functional alterations of vCLC variants represent an obstacle to identifying genotype-phenotype correlations. However, an important piece in the investigation of the entire CLC protein family is still missing: the absence of specific and powerful modulators of CLC protein activity and strategies to improve CLC protein folding, trafficking, and stability. The development of therapeutic approaches is limited by this, and therefore an effort is required to bridge the gap.
